# The relative roles of early life, physical activity, sedentarism and diet in social and economic inequalities in body mass index and obesity risk between 9 and 18

**DOI:** 10.1016/j.ssmph.2023.101499

**Published:** 2023-08-30

**Authors:** Richard Layte, Frances M. Cronin, Sanna Nivakoski, Olivia McEvoy, Ross Brannigan, Debbi Stanistreet

**Affiliations:** a Department of Sociology, Trinity College Dublin, Dublin, Ireland; b School of Population Health, Royal College of Surgeons University of Medicine and Health Sciences in Ireland, Dublin 2, Ireland; c European Foundation for Living and Working Conditions, Dublin, Ireland

**Keywords:** Socioeconomic differentials, BMI, Body mass index, Overweight, Obesity, Maternal education

## Abstract

**Background:**

Studies in many middle and high-income countries describe an increasing prevalence of adiposity and obesity among children and adolescents. Prevalence is higher among families of low socioeconomic position (SEP) and systematic reviews have identified relevant factors, but have not quantified their relative importance to SEP differentials. This paper examines the relative importance of different factors to Body Mass Index (BMI) and obesity trajectories from age 9 to age 17/18.

**Methods:**

Multi-level models of child BMI/obesity risk trajectory by maternal education were conducted using a nationally representative cohort of children born in Ireland in 1998 and aged 9 at baseline (N = 8568), with follow-up at 13 and 17/18 years (88% and 73% response rate respectively). Models were stratified by sex and both time-varying (e.g. child physical activity, diet, sedentary activity) and time-invariant (e.g. early life) factors were tested.

**Results:**

Significant inverse gradients in BMI and obesity risk by level of maternal education were present across both sexes and at each age; unadjusted absolute differentials in obesity risk between highest/lowest education groups increased by 56% for males and 42% for females between age 9 and 17/18. Early life factors accounted for 22% of the differential in obesity risk between the lowest and highest education groups among males at age 9, falling to 13% at 17/18. Among females the proportion fell from 33 to 23%. Unadjusted absolute high/low maternal education group differentials in BMI were 7.5 times higher among males and 11 times higher among females at 17/18 than at age 9.

**Conclusions:**

Given the importance of early life exposures to subsequent differentials in BMI and obesity risk our findings suggest that policy makers should focus resources on primary prevention during the prenatal and early life period if they wish to reduce the prevalence of child and adolescent obesity.

## Highlights

• Systematic review has identified the most important risk factors for Socio-economic inequalities in child obesity.
• Little is known about the relative importance of different risk factors to socio-economic inequalities in child obesity.
• Differentials in obesity risk by maternal education increased by 56% for males and 42% for females between age 9 and 17/18.
• Early life factors accounted for 20% of the high/low differential in obesity risk among males at age 9; 12% at 17/18.
• Among females, early life factors accounted for 32% of the maternal educational differential at age 9; 22% at age 17/18.

## 1 Introduction

The prevalence of overweight and obesity among children and adolescents has increased in recent decades across the majority of medium and high income countries examined (Barriuso et al., 2015; de Onis et al., 2010; NCD-RisC, 2020). Obesity risk has been shown to track from childhood into adulthood and obesity in adulthood is now a leading cause of morbidity and mortality worldwide (WHO, 2013). To date there is weak evidence for the effectiveness of therapeutic interventions for children and adolescents living with obesity (Danielzik et al., 2004; Stamatakis et al., 2010) which would suggest that primary prevention is a more effective policy option.

Socio-economic position (SEP) has been found to be strongly related to the risk of obesity among children (Griffiths et al., 2009; Reilly et al., 2005) and whilst data have indicated a recent plateauing in child obesity trends among children from higher SEP groups within countries (Chung et al., 2016), there is also evidence that prevalence among lower SEP groups continues to rise leading to growing SEP disparities (Stamatakis et al., 2005, 2010).

A child’s risk of overweight and obesity is the result of the complex interaction of different factors from early life. To date, research has highlighted the role of variation in genotype, birth weight, breastfeeding, weaning practices, maternal smoking in pregnancy, maternal consumption of alcohol in pregnancy and child dietary quality and physical activity in overall obesity risk among children and young people (Danielzik et al., 2004; Griffiths et al., 2009; Oken et al., 2007; Reilly et al., 2005; Von Kries et al., 2002) but less is known about the role of these factors for SEP variation in risk of obesity. Recent systematic reviews examining SEP differentials in childhood obesity have identified several modifiable risk factors in early life including infant birthweight, low breastfeeding initiation and duration, early age at weaning, maternal smoking during pregnancy, poor child dietary behaviours (particularly consumption of sugar-sweetened beverages, and not eating breakfast), high child sedentary behaviours and low physical activity and maternal BMI/pre-pregnancy weight (Cameron AJ et al., 2015; Cronin et al., 2022; Gebremariam et al., 2017). However, systematic reviews cannot assess the relative contribution of these different factors to SEP differentials overall, that is, their importance at the population level in accounting for the mean differentials in BMI or risk of obesity between young people in different SEP groups. Without this information it is difficult for policy makers to assess the most effective periods and factors on which to base primary prevention. For example, are risk factors in early life (e.g. prenatal smoking and breastfeeding) more important for child obesity risk compared to later risk factors such as child life-style behaviours (e.g. physical activity and diet)? In response, this paper focuses on the role of different groups of factors in social gradients in body mass index and obesity risk trajectories from middle childhood (age 9) to early adulthood (age 17/18) using data from a nationally representative child cohort study from Ireland. These data allow us to empirically assess the role of prospectively collected measures of physical activity, diet and sedentary behaviours.

## 2 Methods

### 2.1 Sample

The Growing Up in Ireland (GUI) study is a nationally representative, longitudinal study commenced in 2007–2008. The study sample design uses a two-stage sampling process with the primary sampling unit being primary schools within the Republic of Ireland (n = 3200) with children within these schools as the second unit. A probability proportionate to size (PPS) sampling method was used to select 1105 schools, of which 909 agreed to participate (response rate 82%). Within these schools, a random sample of eligible children (aged 9 years at the first interview) were selected (response rate 57%). Of the 8568 9-year-olds (and families) included in the first wave, 7525 families (88%) were followed up at age 13 and 6216 (73%) at age 17/18 years.

### 2.2 Missing data

**Fig. 1:**
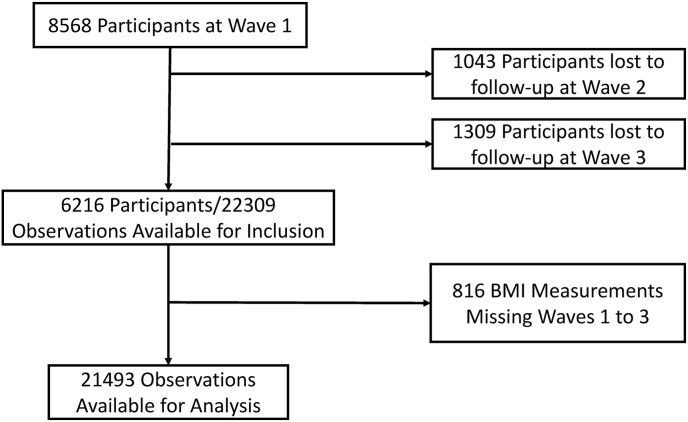
Sample flow chart

The loss of participants to follow-up raises concerns that the pattern of attrition may be selective and thus introduce systematic bias into our analyses. The data were reweighted (Thornton et al., 2010) for analyses to take account of the original sample error and subsequent attrition using a range of factors and a minimum distance algorithm. Of the 22,309 observations at ages 9, 13 and 17/18, data on either/both height and weight were missing for 816 observations (3.7%) with 72 individuals having no valid weight or height measure (0.8%). Observations missing data on height or weight were excluded from the analysis. Maternal education was not a significant predictor of missing a valid measure of BMI (P > 0.05). Levels of non-response across the 21,493 remaining observations varied across variables but was heaviest for our measures of sedentary exposures at 1%. This level of missing data has the potential to bias the analyses. In response, missing values were imputed for all predictor variables using multiple imputation by chained equations as implemented in STATA 17 (Royston, 2004). Fig. 1 provides details of the loss of observations due to attrition to the original sample and non-response.

### 2.3 Dependent variable

Height and weight measurements were made of the study child as part of the household interview by trained interviewers using a SECA 761 medically approved (Class IIII) flat mechanical scale that graduated in 1 kg increments and had an upper capacity of 150 kg. Weight was recorded to the nearest 0.5 kg. Height was recorded to the nearest millimetre using a Leicester portable height stick. Respondents were asked to remove footwear, headwear and any heavy clothing prior to being measured. The data were screened by the GUI data management team for biologically implausible data prior to deposit in the archive and extreme outliers were set to missing. Child BMI was calculated using the child’s measured height and weight at each wave (kg/m^2^) and a cut-off for obesity established using standard sex and age specific thresholds (Cole et al., 2000).

### 2.4 Exposure variable: maternal education

Level of maternal education was used as a measure of household social and economic group. Maternal education captures the knowledge-related assets and health literacy of the mother which is influential in shaping the probability of the child engaging in health-compromising behaviours (Devaux & Sassi, 2013; Roskam & Kunst, 2008). Maternal, rather than paternal, education was chosen as some studies have documented higher correlations of childhood BMI with maternal as opposed to paternal BMI, and some researchers suggest that the maternal environment (particularly the intrauterine environment) may play a central role in determining children's BMI trajectories (Kramer et al., 2000; Lawlor et al., 2007). Measured at baseline, maternal education was categorised into four groups using the International Standard Classification of Education (ISCED) (Zelener & Schneider, 2016): lower secondary level education or less (ISCED <2.2), upper secondary education (ISCED 2.2-2.3), post-secondary (ISCED 3-3.03) and degree or higher (ISCED 4+).

### 2.5 Mediating variables

#### 2.5.1 Early life factors

High birthweight is well established as a predictor of overweight and obesity (Danielzik et al., 2004). Birthweight derived from birth records is analysed in categorical form (<2.5 kg; 2.5–3 kg; 3–4.5 kg; 4.5 + kg). Systematic reviews have found repeated associations between maternal smoking and alcohol consumption in pregnancy and SEP gradients in risk of overweight and obesity (Gebremariam et al., 2017). We use maternal report of consumption of alcohol (never; occasionally; weekly or more) and cigarettes (never; less than daily; 1–5; 6–10; 11–25; 26+ daily) in pregnancy. Maternal reported breastfeeding duration is categorised as never, <3 months’, 3–6 months, 6+ months.

#### 2.5.2 Physical activity

Physical activity levels were calculated using the Godin-Shephard Leisure Time Exercise Questionnaire (Godin & Shephard, 1985). PCG reported the number of days in the previous 14 that the young person engaged in “hard” or “light” exercise (separately) for at least 20 min. Hard exercise was defined as “enough to make him/her breathe heavily and make his/her heart faster”. Light exercise was defined as “not hard enough to make him/her breathe heavily and make his/her heart beat fast” (Godin & Shephard, 1985). The Godin-Shephard has previously demonstrated concurrent validity with measures of maximum oxygen intake and muscular endurance (Godin et al., 1986), construct validity (Godin & Shephard, 1985) and test-retest reliability (Sallis et al., 1993; Zelener & Schneider, 2016). In addition, at each wave, the young person was asked “how often do you do each of these activities?” with two of the subsequent options being “A. Play sports or undertake physical activities without a coach or instructor (e.g. biking, skate-boarding etc.)?” and “B. Play sports with a coach or instructor, or as part of an organised team, other than in P.E. class? (swimming, soccer, hockey, etc)?” (never, less than weekly, several times a week). Physical activity as part of commute to school was assessed at each wave through parental response to the question “How does [Study Child] usually go to school?” (walking/cycling, car, bus).

#### 2.5.3 Sedentary activities

At each wave the young person was asked how much time they spend doing each of the following: watching TV, interacting online (social media/browsing), computer gaming using the question stem “On a normal weekday during term-time, about how much time do you spend … ?”. Responses were coded into categories: none, less than an hour, 1 h to less than 3 h, 3 h to less than 5 h, 5 h to less than 7 h, 7 h or more.

#### 2.5.4 Dietary quality

Dietary quality was characterised using the Amhurst Sallis Food Frequency Questionnaire by asking the young person how often they had eaten thirteen different food types in the previous 24 h at each wave (never/once/more than once). Fresh fruit, cooked or raw vegetable and cheese, yogurt and other dairy products were defined as ‘heathy’. Processed meats (e.g. sausages hamburgers), fried foods (e.g. chips/crisps) and sweet deserts (e.g. biscuits, donuts, cake) were defined as ‘unhealthy’. ‘Healthy’ foods were scored positively (once = 1; more than once = 2) and ‘unhealthy’ foods negatively. Summing the food types yields a scale which varies between -12 and 12+ at each wave and provides a time-varying dietary quality score. Given the importance of consumption of breakfast and sugar sweetened drinks in recent systematic reviews for SEP gradients in obesity (Cronin et al., 2022; Gebremariam et al., 2017), measures of whether the participant consumed sweetened beverages in the last 24 h (no, once, 2+) and usually eats breakfast (yes, no) are included in the analysis.

## 3 Analysis

Multi-level linear spline models (Howe et al., 2016), with appropriate study weights, were used to estimate body mass index/obesity probability trajectory differentials by maternal education between ages 9 and 17/18. To account for the nesting of observations within individuals, each model includes a random intercept (μ_0j_) for each young person, which follows a bivariate normal distribution and measurement error is explicitly modelled over time (e_0i_j). The random intercepts model for linear change in body mass index/obesity probability can be written as:

Yi_j_ = β_0_ + β_1_ x (Age)_ij_ + β_2_ x (Maternal education)_j_ + β_3_ x (Maternal education)_i_ x (Age)_jj_ + X_ij1_ … _n_ + X_j1_ … _n_ + μ_0j+_e_0ij_

Here, β_0,_ β_1,_ β_2_ and β_3_ are ‘fixed’ coefficients which represent the average intercept across the sample and the average slopes for age, maternal education and the interaction of age and maternal education respectively. The interaction of age and maternal education are included in the model to allow the effect of age to vary across levels of maternal education. X_ij1 … n_ represents a vector of time varying mediating variables (i.e. physical activity, sedentary activities, dietary quality) and X_j1 … n_ a vector of time invariant mediating variables (i.e. maternal education and early life factors). μ_0j_ is a ‘random’ coefficient which represents the individual child’s deviation from the average intercept. e_0ij_ represents the observation-level measurement error. We quantify the role of different factors in accounting for differentials in body mass index/obesity probability using mediation analysis utilising the difference method (VanderWeele, 2016). Here, the contribution of different mediating processes to maternal education differentials is quantified by comparing the slope coefficients in a reference model to those after adjusting for the mediating process of interest. We quantify the role of different groups of predictors in accounting for the maternal education differential by calculating the change in β_1_+β_2_+β_3_ with the addition of each group of predictors to a ‘base model’. Models were stratified by sex and all analyses were performed in Stata/SE 17. P-values less than 0.05 were considered statistically significant. Theoretical justification was used to assign variables to groups representing each process of interest:

**Model 1 - Base Model:** Maternal Education + Child Age + Interaction of Education & Age.
**Model 2 - Early life Factors:** Model 1 + Child birth weight; maternal smoking in pregnancy; maternal alcohol consumption in pregnancy; whether child was breastfed.
**Model 3 - Physical Activity:** Model 1 + Whether child plays sport; active commute to school; overall physical activity (Godin Shepherd)
**Model 4 - Sedentary Behaviors:** Model 1 + Child time online; child time watching TV; child time gaming.
**Model 5 - Dietary Factors**: Model 1 + Whether child usually eats breakfast; child consumption of sugar sweetened drinks; overall dietary quality (Amhurst Sallis).
**Model 6** – **Fully Adjusted** (Base + Early Life + Physical Activity + Sedentary Behaviours + Dietary Factors)

## 4 Results

At each wave of data collection, children of all maternal education groups had significantly higher mean BMIs relative to the children of degree level educated mothers and had a higher probability of being defined as obese (P < 0.05). Unadjusted absolute differentials in obesity risk between lowest/highest education groups increased by 56% between 9 and 17/18 for males and 42% among females. Unadjusted absolute high/low differentials in BMI were 7.6 times higher among males and 11.7 times higher among females at 17/18 than at age 9. Children and young people whose mothers had less than degree level education were on average significantly more likely to have higher levels of the risk factors for obesity as identified by recent systematic reviews (Cronin et al., 2022; Gebremariam et al., 2017) in bivariate analyses (see Table 1): higher levels of low birthweight (<2.5 kg), higher exposure to prenatal smoking, lower levels of breastfeeding, less physical activity, higher sedentary activity (TV, online time, gaming), a lower quality diet, eating breakfast less regularly and higher consumption of sweetened drinks. The only exceptions to this pattern were a higher exposure to alcohol in pregnancy and a lower probability of active commute to school among the children and young adults of degree level mothers.

**Table 1:** Means/proportions of dependent and independent variables (weighted) by level of maternal education.

Variable		Unweighted N	All	Maternal Education			
				Lower 2nd or Less	Upper 2nd	Post Secondary	Degree or Higher
Weight Status	% Healthy	16,179	73.3	**66.8**	**72.9**	**76.1**	79.4
	% Overweight	4104	20.1	**22.3**	**21.1**	18.4	17.2
	% Obese	1210	6.6	**11.0**	**6.0**	**5.5**	3.5
	Mean Body Mass Index at 9	8568	**18.0**	**18.4**	**17.9**	17.8	17.5
	Mean Body Mass Index at 13	7525	**20.8**	**21.8**	**20.9**	20.5	20.2
	Mean Body Mass Index at 17/8	6216	**23.2**	**24.1**	23.2	22.9	22.7
Birth Weight	% <2.5 kg	373	4.8	**6.4**	4.3	3.8	3.9
	% 2.5–3 kg	916	11.6	**15.1**	10.0	10.1	10.5
	% 3–4.5 kg	6769	79.0	**74.4**	81.2	81.4	80.0
	% >4.5 kg	392	4.6	4.1	4.5	4.7	5.6
Maternal Smoking in Pregnancy	% None	6402	71.9	**52.9**	**76.3**	**79.8**	88.3
	% Occasionally	787	11.3	**15.3**	**10.4**	**11.0**	6.4
	% 1–5 Daily	169	2.5	**4.0**	**2.1**	1.7	1.1
	% 6–10 Daily	469	7.6	**13.8**	**6.3**	**5.0**	1.9
	% 11–25 Daily	380	6.2	**12.2**	4.7	2.5	2.2
	% 26+ Daily	27	0.6	**1.7**	0.2	0.0	0.1
Maternal Alcohol Consumption in Pregnancy	% None	5021	63.2	**67.5**	**64.7**	**60.1**	55.1
	% Occasionally	3091	35.3	**30.9**	**33.8**	**38.6**	43.4
	% Weekly or More	116	1.5	1.6	1.6	1.3	1.5
Was Child Breastfed?	% No	4232	56.2	**77.1**	**58.3**	**43.5**	27.1
	% <3 Months	2356	24.3	**14.1**	**25.1**	31.4	34.0
	% 3–6 Months	987	9.4	**4.4**	**7.9**	**11.4**	19.5
	% 6+ Months	993	10.1	**4.4**	**8.7**	**13.8**	19.4
Does Young Person Play Sport?	% No	2835	12.8	**15.8**	**15.2**	**11.1**	9.7
	% Less Than Weekly	3792	17.1	**22.7**	**17.7**	**16.8**	14.0
	% Weekly	7017	31.6	**27.0**	**30.9**	33.2	33.3
	% Several Times Week	8577	38.6	**34.5**	**36.2**	**38.9**	43.0
Young Person Commute to School	% Walking or Cycling	3728	23.2	**31.8**	22.6	21.1	21.1
	% Bus	3370	20.9	21.2	**21.5**	21.4	19.8
	% Car	8994	55.9	**47.0**	**55.9**	57.5	59.1
Physical Activity	Mean Godin Shephard Scale	22,219	91.0	**88.3**	**87.7**	**92.8**	94.4
Sedentary Activities	Mean Hours Watching TV	22,128	2.85	**3.05**	**2.93**	**2.86**	2.67
	Mean Hours Online	22,134	2.59	**2.54**	**2.69**	2.53	2.56
	Mean Hours Gaming	22,139	1.77	**1.94**	**1.83**	**1.76**	1.65
Dietary Quality	Mean Amhurst Sallis Scale	22,309	1.60	**0.39**	**1.31**	**1.73**	2.39
Does Child Have Breakfast?	% No	1756	7.9	**11.6**	**9.4**	**6.8**	5.2
	% Yes	20,552	92.1	**88.4**	**90.6**	**93.2**	94.8
Does Child Consume Non-Diet Carbonated Drinks?	% No	12,491	56.3	**42.2**	**54.3**	**57.0**	64.4
	% Once Last 24 Hours	5823	26.2	**30.7**	**27.0**	25.9	23.6
	% Twice or More Last 24 Hours	3892	17.5	**27.1**	**18.7**	**17.2**	12.0

Key: Bolded figures are significantly different from compared to the reference category of degree or higher.

Table 2a shows that mean BMI increases significantly with age for both sexes and that the growth trajectory is steeper for the children of mothers with the lowest level of education after adjustment for the full set of mediating variables (as shown by the interaction of age and maternal education). Table 2a also shows that, whereas low birthweight significantly reduces mean BMI, birthweights higher than 4.5 kg are associated with a higher mean BMI across both sexes (P < 0.001). High birthweight is also associated with a significant increase in the probability of obesity for both male and female participants (P < 0.01) (Table 2b). Maternal smoking in pregnancy continued to be positively associated with both higher average BMI and obesity risk adjusting for other factors, particularly among female participants where there is a notable dose response pattern with the level of reported daily smoking in pregnancy. Less breastfeeding in infancy (compared to 6+ months) and absence of breastfeeding in particular, are associated with significantly higher BMI but were not a significant predictor of obesity risk, except among female participants where absence of breastfeeding in infancy was associated with an increase of 1.18 in the log odds of obesity (P < 0.001). Frequency of participation in sport did not remain a significant predictor of child BMI after adjustment, but is associated with a significantly lower risk of obesity among female participants (several times a week: -0.54, P = 0.02) and overall physical activity, as measured using the Godin Shepard Scale remained a significant negative predictor of both mean BMI and obesity risk (P < 0.001). Dietary quality is not a significant predictor of BMI or obesity but regular consumption of breakfast is associated with a significantly lower BMI (P < 0.01) and obesity risk (<0.001), although the latter was only found among female participants).

**Table 2a:** Coefficients, t-statistics and P Values for the Fully Adjusted Linear Spline Model of Body Mass Index by Sex.

		Male Fully Adjusted Model					Female Fully Adjusted Model				
		Coeff.	t-stat	P value	-95%CI	+95%CI	Coeff.	t-stat	P value	-95%CI	+95%CI
Age (Ref Age 9)	Age										
	13	2.13	21.12	<0.001	1.93	2.33	2.94	22.76	<0.001	2.68	3.19
	17/8	4.72	38.14	<0.001	4.48	4.97	4.79	29.43	<0.001	4.47	5.11

Maternal Highest Education (Ref: Third Level)											
	Lower 2nd	0.48	3.14	0.002	0.18	0.77	0.18	1.04	0.297	-0.16	0.52
	Higher 2nd	0.22	1.69	0.09	-0.03	0.47	0.13	0.84	0.403	-0.17	0.43
	Post Secondary	0.06	0.44	0.659	-0.20	0.32	0.14	0.9	0.37	-0.17	0.46

Wave * Maternal Education Interaction											
	Age 13 * Lower 2nd	0.49	3.11	0.002	0.18	0.80	0.53	3.01	0.003	0.18	0.87
	Age 13 * Higher 2nd	0.19	1.52	0.129	-0.06	0.44	0.13	0.84	0.401	-0.17	0.43
	Age 13 * Post Secondary	0.16	1.13	0.26	-0.12	0.43	-0.03	-0.15	0.879	-0.35	0.30
	Age 17/8 * Lower 2nd	0.48	2.69	0.007	0.13	0.83	0.72	3.56	<0.001	0.32	1.12
	Age 17/8 * Higher 2nd	0.10	0.67	0.505	-0.19	0.38	0.35	1.98	0.048	0.00	0.70
	Age 17/8 * Post Secondary	0.21	1.23	0.218	-0.12	0.54	0.13	0.61	0.541	-0.28	0.53
Birth Weight (Ref: 3 kg–4.5 kg)											
	<2.5 kg	-0.24	-1.06	0.287	-0.70	0.21	-0.67	-2.77	0.006	-1.15	-0.20
	2.5–3 kg	-0.45	-2.82	0.005	-0.76	-0.14	-0.49	-3.24	0.001	-0.79	-0.19
	>4.5 kg	0.57	3.01	0.003	0.20	0.95	1.14	3.97	<0.001	0.58	1.70

Maternal Smoking in Pregnancy (Ref: None)											
	Occasionally	0.40	2.63	0.009	0.10	0.69	0.51	2.74	0.007	0.14	0.88
	1-5 Daily	0.47	1.26	0.21	-0.27	1.22	0.90	2.63	0.009	0.22	1.57
	6-10 Daily	0.36	1.58	0.119	-0.09	0.80	0.73	3.25	0.001	0.29	1.18
	11-25 Daily	0.45	1.91	0.057	-0.01	0.92	0.92	3.93	<0.001	0.46	1.37
	26+ Daily	-0.87	-1.03	0.303	-2.54	0.80	1.80	2.1	0.036	0.12	3.48

Maternal Alcohol Consumption in Pregnancy (Ref: None)											
	Occasionally	-0.31	-3.41	0.001	-0.49	-0.13	-0.36	-3.36	0.001	-0.57	-0.15
	Weekly or More	0.06	0.15	0.883	-0.71	0.82	-0.25	-0.56	0.575	-1.11	0.62

Was Child Breastfed? (Ref: 6+ Months)											
No	No	0.43	2.83	0.005	0.13	0.73	0.66	4.06	<0.001	0.34	0.99
<3 Mnths	<3 Mnths	0.39	2.48	0.013	0.08	0.70	0.32	1.89	0.059	-0.01	0.66
3-6 Mnths	3-6 Mnths	0.07	0.36	0.721	-0.30	0.43	0.44	2.17	0.03	0.04	0.84

Does Young Person Play Sport? (Ref: No)											
	Less Than Weekly	0.10	0.96	0.339	-0.10	0.29	0.05	0.6	0.546	-0.11	0.21
	Weekly	0.10	1.15	0.251	-0.07	0.28	-0.03	-0.34	0.733	-0.18	0.13
	Several Times Week	0.04	0.49	0.627	-0.13	0.22	-0.07	-0.89	0.373	-0.24	0.09

Young Person Commute to School (Ref: Walking or Cycling)											
	Bus	0.10	1.29	0.196	-0.05	0.26	0.02	0.28	0.779	-0.15	0.20
	Car	0.11	1.66	0.096	-0.02	0.25	-0.06	-0.83	0.406	-0.21	0.08

Physical Activity	Godin Shephard Scale	-0.01	-5.16	<0.001	0.00	0.00	0.00	-2.07	0.038	0.00	0.00
Young Person Sedentary Activities											
	Time Watching Television	0.07	2.77	0.006	0.02	0.13	0.08	2.78	0.005	0.02	0.13
	Time Online	0.00	0.16	0.874	-0.04	0.05	0.04	1.63	0.104	-0.01	0.09
	Time Gaming	0.03	1.37	0.170	-0.01	0.08	0.07	2.03	0.043	0.00	0.14
Young Person Dietary Quality	Amhurst Sallis Scale	0.00	-0.1	0.920	-0.05	0.04	0.02	0.87	0.385	-0.03	0.08
Does Child Have Breakfast? (Ref: No)	Yes	-0.30	-3.24	0.001	-0.48	-0.12	-0.34	-4.04	<0.001	-0.51	-0.18

Does Child Consume Non-Diet Carbonated Drinks? (Ref: No)											
	Once Last 24 Hours	0.07	1.29	0.197	-0.04	0.17	0.05	0.91	0.362	-0.06	0.17
Twice or More Last 24 Hours		0.07	1.13	0.260	-0.05	0.20	-0.02	-0.24	0.809	-0.16	0.13

Constant		17.50	75.48	<0.001	17.04	17.95	17.50	70.13	<0.001	17.01	17.99
N Observations			10,652					11,200			
N Individuals			4150					4381			
Within Individual Variance			2.57					2.99			
Between Individual Variance			1.75					1.92			
% Variance within Individuals			0.68					0.71

**Table 2b:** Coefficients, t-statistics and P Values for the Fully Adjusted Logistic Spline Model of Obesity Risk by Sex.

		Male Fully Adjusted Model					Female Fully Adjusted Model				
		Coeff.	t-stat	P value	-95%CI	+95%CI	Coeff.	t-stat	P value	-95%CI	+95%CI
Age (Ref Age 9)	Age										
	13	-0.33	-0.79	0.432	-1.16	0.50	-0.31	-0.69	0.491	-1.195	0.573
	17/8	-0.41	-0.8	0.422	-1.42	0.60	0.10	0.19	0.852	-0.939	1.137

Maternal Highest Education (Ref: Third Level)											
	Lower 2nd	1.18	2.56	0.011	0.28	2.08	0.93	2.08	0.037	0.056	1.811
	Higher 2nd	0.66	1.54	0.124	-0.18	1.51	0.86	1.97	0.049	0.003	1.709
	Post Secondary	0.15	0.32	0.747	-0.77	1.07	0.65	1.4	0.161	-0.258	1.549

Wave * Maternal Education Interaction											
	Age 13 * Lower 2nd	0.16	0.3	0.761	-0.89	1.22	0.10	0.18	0.855	-0.945	1.140
	Age 13 * Higher 2nd	-0.20	-0.4	0.687	-1.17	0.77	-0.53	-1.03	0.304	-1.531	0.477
	Age 13 * Post Secondary	-0.41	-0.72	0.472	-1.53	0.71	-0.39	-0.72	0.473	-1.465	0.680
	Age 17/8 * Lower 2nd	1.22	2.02	0.043	0.04	2.40	0.01	0.02	0.986	-1.131	1.151
	Age 17/8 * Higher 2nd	0.50	0.88	0.380	-0.61	1.61	-0.74	-1.33	0.183	-1.837	0.350
	Age 17/8 * Post Secondary	0.70	1.05	0.293	-0.60	2.00	-0.63	-0.98	0.329	-1.889	0.633

Birth Weight (Ref: 3 kg–4.5 kg)											
	<2.5 kg	0.53	1.18	0.237	-0.35	1.42	-0.83	-1.72	0.085	-1.769	0.113
	2.5–3 kg	-0.38	-1.06	0.290	-1.09	0.33	-0.77	-2.51	0.012	-1.379	-0.171
	>4.5 kg	0.96	2.62	0.009	0.24	1.68	1.49	3.4	0.001	0.633	2.352

Maternal Smoking in Pregnancy (Ref: None)											
	Occasionally	0.11	0.32	0.753	-0.56	0.77	0.56	1.66	0.098	-0.103	1.216
	1-5 Daily	-0.08	-0.1	0.921	-1.57	1.42	0.97	1.83	0.068	-0.072	2.021
	6-10 Daily	0.97	2.59	0.010	0.24	1.71	0.91	2.49	0.013	0.193	1.632
	11+ Daily	0.11	0.25	0.803	-0.77	1.00	1.06	2.88	0.004	0.340	1.790
Maternal Alcohol Consumption in Pregnancy (Ref: None)											
	Occasionally	-0.54	-2.45	0.014	-0.97	-0.11	-0.48	-2.46	0.014	-0.866	-0.099
	Weekly or More	0.87	1.23	0.219	-0.51	2.25	-0.34	-0.45	0.65	-1.802	1.125

Was Child Breastfed? (Ref: 6+ Months)											
No	No	0.77	2	0.045	0.02	1.53	1.17	3.33	0.001	0.482	1.862
<3 Mnths	<3 Mnths	0.59	1.46	0.145	-0.20	1.38	0.47	1.25	0.211	-0.266	1.200
3-6 Mnths	3-6 Mnths	0.53	1.13	0.259	-0.39	1.46	0.74	1.72	0.085	-0.102	1.584

Does Young Person Play Sport? (Ref: No)											
	Less Than Weekly	0.32	1.09	0.277	-0.26	0.90	-0.30	-1.32	0.186	-0.744	0.145
	Weekly	-0.13	-0.47	0.642	-0.65	0.40	-0.45	-2.15	0.032	-0.864	-0.039
	Several Times Week	-0.41	-1.52	0.128	-0.94	0.12	-0.55	-2.4	0.016	-1.008	-0.102

Young Person Commute to School (Ref: Walking or Cycling)											
	Bus	0.29	1.21	0.227	-0.18	0.77	0.19	0.87	0.382	-0.240	0.626
	Car	0.20	0.95	0.342	-0.22	0.62	-0.05	-0.28	0.779	-0.431	0.323

Physical Activity	Godin Shephard Scale	-0.01	-6.63	<0.001	-0.02	-0.01	-0.01	-3.34	0.001	-0.010	-0.003
Young Person Sedentary Activities											
	Time Watching Television	0.17	2.01	0.045	0.00	0.34	0.09	1.22	0.224	-0.058	0.246
	Time Online	-0.19	-2.36	0.018	-0.35	-0.03	-0.03	-0.4	0.693	-0.171	0.113
	Time Gaming	0.32	4.23	<0001	0.17	0.46	0.21	2.4	0.016	0.038	0.377
Young Person Dietary Quality	Amhurst Sallis Scale	0.09	0.89	0.374	-0.10	0.27	-0.12	-1.15	0.251	-0.323	0.084
Does Child Have Breakfast? (Ref: No)	Yes	-0.48	-1.82	0.069	-0.99	0.04	-0.88	-4.4	0	-1.274	-0.489

Does Child Consume Non-Diet Carbonated Drinks? (Ref: No)											
	Once Last 24 Hours	0.31	1.75	0.079	-0.04	0.66	0.06	0.38	0.704	-0.265	0.393
Twice or More Last 24 Hours		0.14	0.63	0.526	-0.29	0.57	-0.04	-0.21	0.837	-0.444	0.360

Constant		-5.98	-7.99	<0001	-7.45	-4.52	-6.29	-9.56	<0001	-7.578	-5.000
N Observations			10,652					11,200			
N Individuals			4150					4381			
Within Individual Variance			2.23	0.12				2.63	0.06		
Between Individual Variance			3.04	0.18				3.73	0.12		
% Variance within Individuals			0.74	0.02				0.81	0.01		

The proportionate reduction in maternal educational differential varied significantly by sex, dependent variable (BMI or obesity risk), age (9, 13 or 17) and model tested (base, early life, sedentary, physical, diet or fully adjusted). The fully adjusted models account for between 33% and 65% of the educational differential in BMI for both males and females at age 9. The success of the fully adjusted female model of BMI fell quickly by age to 11% or less at 17/18. This pattern was common to male and female models of BMI, which were more successful at accounting for educational differentials at 9 than at later ages. The fully adjusted model of male and female BMI differentials accounted for <9% of the differential at age 17/18 (see Table 3a).

**Table 3a:** Proportionate reduction in maternal education differential relative to the by variable group over base model variables by young Person’s age and sex – linear spline models of body mass index.

	Maternal Highest Education	Early Life	Sedentary	Physical	Diet	Fully Adjusted
		Male – Reference: Degree or Above				
	Lower Secondary	24%	3%	2%	5%	33%
Age 9	Upper Secondary	30%	3%	2%	10%	42%
	Post Secondary	41%	6%	4%	10%	65%

	Lower Secondary	5%	2%	4%	2%	12%
Age 13	Upper Secondary	4%	1%	4%	3%	12%
	Post Secondary	3%	1%	4%	2%	10%

	Lower Secondary	3%	1%	1%	1%	5%
Age 17/8	Upper Secondary	2%	0%	2%	3%	8%
	Post Secondary	1%	0%	2%	2%	5%
		Female – Reference: Degree or Above				
	Lower Secondary	67%	3%	-1%	2%	65%
Age 9	Upper Secondary	66%	6%	0%	-8%	57%
	Post Secondary	52%	6%	-4%	-14%	33%

	Lower Secondary	9%	2%	2%	1%	12%
Age 13	Upper Secondary	6%	1%	2%	0%	8%
	Post Secondary	4%	2%	2%	0%	6%

	Lower Secondary	6%	2%	2%	-1%	7%
Age 17/8	Upper Secondary	4%	2%	2%	1%	6%
	Post Secondary	2%	2%	2%	0%	5%

**Fig. 2:**
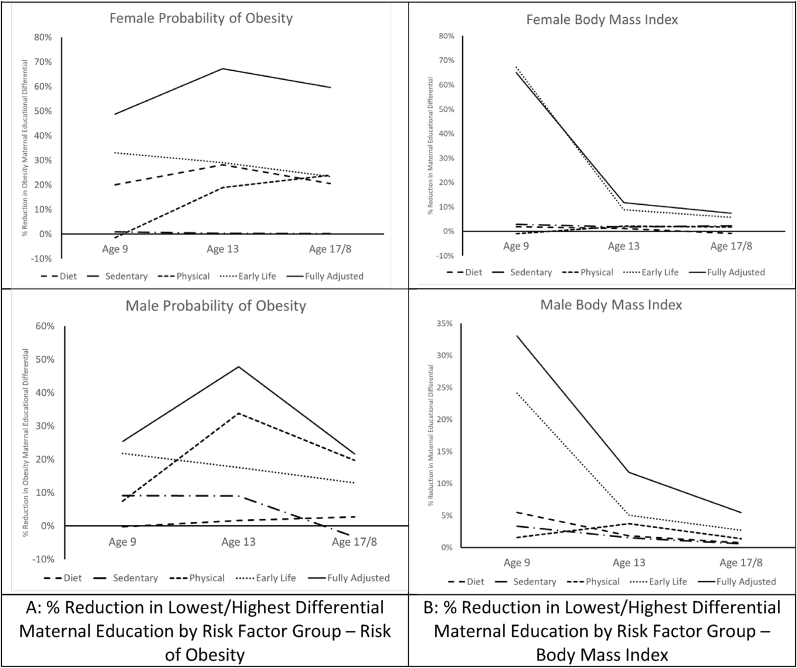
% reduction in lowest/highest differential maternal education by risk factor group, sex and weight status measure.

Our models of obesity risk accounted for more of the maternal educational differential than the models of BMI and, in contrast to the latter, the fully adjusted models were more successful at accounting for differentials at 13 and 17/18 than at age 9 for both male and female participants (see Table 3b). If we focus on the differential between the lowest and highest educational groups (see Fig. 2) the different groups of risk factors varied in their ability to account for educational differentials. Among female participants, early life factors accounted for a higher proportion of the differential in both BMI and obesity risk differentials at each age compared to all other factors. Among males, this pattern only applied in the models of BMI: early life factors accounted for more of the differential in BMI at all ages compared to other groups of factors. For obesity risk among males on the other hand, the model of physical activity behaviours accounted for more of the differential at ages 13 and 17/18 (see Fig. 2, Panel A). Compared to males, physical activity and dietary factors accounted for more of the educational differential among females, although less than early life factors.

**Table 3b:** Proportionate reduction in maternal education differential by variable group over base model by young Person’s age and sex – logistic spline models of obesity risk.

	Maternal Highest Education	Early Life	Sedentary	Physical	Diet	Fully Adjusted
		Male – Reference: Degree or Above				
	Lower Secondary	22%	9%	7%	0%	25%
Age 9	Upper Secondary	27%	14%	9%	-14%	16%
	Post Secondary	42%	18%	2%	11%	54%

	Lower Secondary	18%	9%	34%	2%	48%
Age 13	Upper Secondary	27%	12%	66%	3%	84%
	Post Secondary*	52%	24%	169%	18%	207%

	Lower Secondary	13%	-3%	20%	3%	22%
Age 17/8	Upper Secondary	18%	-13%	43%	-2%	34%
	Post Secondary	26%	-45%	103%	-58%	5%
		Female – Reference: Degree or Above				
	Lower Secondary	33%	1%	-1%	20%	49%
Age 9	Upper Secondary	25%	1%	-1%	18%	40%
	Post Secondary	18%	0%	-2%	28%	45%

	Lower Secondary	29%	0%	19%	28%	67%
Age 13	Upper Secondary	36%	-1%	28%	46%	98%
	Post Secondary	30%	-1%	35%	54%	107%

	Lower Secondary	23%	0%	24%	20%	60%
Age 17/8	Upper Secondary	23%	-3%	36%	40%	86%
	Post Secondary	19%	-8%	46%	40%	90%

*A non-significant negative coefficient for the interaction of Age 13*post-secondary (see Table 2b) has been set to zero when calculating these reductions for clarity.

## 5 Discussion

This study contributes to the growing literature on socio-economic inequalities in child and adolescent obesity risk. We find significant inverse gradients in BMI and obesity risk by maternal education across both sexes and at each age between 9 and 17/18. As found in previous literature, this study also shows that socio-economic differentials in BMI and obesity risk increase in adolescence (McCrory et al., 2019). A central objective of this paper was the quantification of the contribution of different risk factors to socio-economic differentials. Our results indicated that early life factors accounted for the largest proportion of the differential in obesity risk compared to other risk factors. However, physical activity and diet were still important in accounting for differentials among female participants at each age and were the most important factor for males after childhood. Our fully adjusted models of body mass index trajectory accounted for between 33 and 65% of both the male and female maternal educational differentials at age 9, but this proportion fell to between 5 and 8% at 17/18. In contrast, our models of obesity risk were more successful at accounting for the educational differential at 13 and 17/18, accounting for up to 90% of risk differentials. Our results add to accumulating evidence that early life factors play an important role in SEP differentials in obesity risk in the child/adolescent population (Sun et al., 2020). A recent multinational, cross-sectional study of almost 5000 children aged 9–11 years from 12 countries, found that breastfeeding was a protective factor for later childhood obesity (Ma et al., 2020) and an earlier study (using this GUI cohort) confirmed a dose-response effect for breastfeeding: compared to those never breastfed, the risk of obesity at age 9 was reduced by 38% for those breastfed for between 13 and 25 weeks, and just over 50% for those breastfed for 26 weeks or more (McCrory & Layte, 2012). Similarly, maternal prenatal smoking continues to be associated with later obesity for the offspring: a study of over 250,000 adults showed that those whose mother smoked during pregnancy had an increased risk of obesity and a reduced likelihood of transitioning from high to normal body weight in adulthood (Sun et al., 2020). Our results raise important questions about why early life factors contribute disproportionately to child and adolescent obesity risk. Prenatal and early life exposures may be causally related to later obesity. If so, this requires a mechanism through which the effects of early environment are conserved and then influence later biological processes and evidence is building that epigenetic processes may be important. Both epigenome wide association studies (Obermann-Borst et al., 2013; Sherwood et al., 2022) and experimental evidence (Paparo et al., 2019) suggest an epigenetic mechanism may link the duration of breastfeeding to subsequent child obesity. Similarly, there is evidence that maternal smoking in pregnancy leads to epigenetic change in off-spring but a causal relationship is yet to be established between prenatal smoking and child obesity (Rogers, 2019). Alternatively, the disproportionate importance of early life exposures in later outcomes may reflect methodological shortcomings in current studies of SEP differentials. Children who experience early life risk factors for obesity (e.g. maternal smoking in pregnancy and the absence of breastfeeding) are also likely to be those that have later lifestyle patterns which contribute to the risk of adolescent overweight and obesity. Adjusting for earlier and later risks using time-varying measures provides a partial answer but the observational nature of the study makes it very difficult to establish a credible counterfactual through which to estimate the independent effects of early life exposures compared to later environment.

Our finding that childhood/adolescent diet and physical activity account for more of the maternal education differential in adolescence compared to childhood suggests a third possible alternative with the influence of lifestyle factors increasing as the child ages, perhaps in combination with a decreasing influence of early life exposures. A recent Cochrane review found that interventions which focus on increasing physical activity were not effective at reducing the risk of obesity before middle childhood, but were effective in adolescence. The review also found no evidence that interventions which focus on diet alone were effective at any point in childhood or adolescence (Brown et al., 2019).

### 5.1 Strengths and limitations

Our analyses are based on high quality, prospectively collected data from a large sample of children and adolescents utilising physical measurements of participant height and weight over almost a decade. The initial sample was nationally representative and any selection bias introduced through initial sample error or subsequent attrition was addressed through statistical weighting. Non-response in the initial sample and differential attrition at follow-up reduced the socio-economic representativeness of the sample as higher income, more educated and employed parents were more likely to respond. Sophisticated inverse probability weights were used to correct for this non-response but it is possible that bias remained. If so this would have the effect of decreasing the magnitude of inequalities in our results.

The availability of a high quality measure of maternal education plus a wide variety of lifestyle behaviours means that we can quantify the role of different groups of factors in differentials, which has not been possible to date. We acknowledge that our measures of early life, diet, physical activity and sedentary behaviours are either parent or child self-report and not based on objective measurements which could give rise to bias. However, we should stress that the measures used are based on internationally validated measures and we would argue that it seems reasonable to assume that any biases which have been introduced through self-report are equal across groups defined by maternal level of education. The focus of this research was the relative differences between groups in different risk factors rather than their absolute levels. The finding that dietary factors account for relatively little of the education differential is in line with the literature, but it is also possible that it is attributable to the measure of dietary quality used which is based on only twenty items and not a full food frequency questionnaire. Similarly, the measure of sedentary behaviours used does not include behaviours which are not computer mediated such as reading a book or other crafts and hobbies. Nonetheless, these variables are consistently measured from middle childhood to early adulthood.

## 6 Conclusion

Given the contribution of obesity to subsequent morbidity and mortality risk, the social patterning of obesity among children and the fact that this increases strongly between 9 and 17/18, may well contribute to widening differentials in mortality risk by SEP in the years to come. Our findings suggest that policy makers should focus resources on primary prevention during the prenatal and early life period if they wish to reduce the prevalence of child and adolescent obesity. However, the pattern of socio-economic inequalities also suggests that public health interventions should also take into account the role of wider societal structures of advantage and disadvantage.

## Research ethics

The study received ethical approval from the Research Ethics Committee of the Office for the Minister for Children and Youth Affairs in Ireland. This study reports results from analysis of deidentified publicly available survey data and is exempt from institutional review board review as per section 46.10(b)of National Institutes of Health document CFR 46.

## Financial disclosure

This study was funded by Health Research Board (HRB) Grant SDAP-2019-026.

## CRediT authorship contributions statement

R.Layte: conceptualization, data curation, methodology and analysis, writing – original draft. F.Cronin: writing – original draft. R.Brannigan: methodology and analysis. D.Stanistreet: writing – review & editing. O.McEvoy: writing – review & editing. S.Nivakoski: writing – review & editing.

## Declaration of competing interests

The authors declare that they have no competing interests.

## Data Availability

Data will be made available on request.
